# Clinical outcomes following robotic versus conventional DIEP flap in breast reconstruction: A retrospective matched study

**DOI:** 10.3389/fonc.2022.989231

**Published:** 2022-09-14

**Authors:** Min Jeong Lee, Jongmin Won, Seung Yong Song, Hyung Seok Park, Jee Ye Kim, Hye Jung Shin, Young In Kwon, Dong Won Lee, Na Young Kim

**Affiliations:** ^1^ Department of Anesthesiology and Pain Medicine, Anesthesia and Pain Research Institute, Yonsei University College of Medicine, Seoul, South Korea; ^2^ Department of Plastic and Reconstructive Surgery, Institute for Human Tissue Restoration, Yonsei University College of Medicine, Seoul, South Korea; ^3^ Department of Surgery, Yonsei University College of Medicine, Seoul, South Korea; ^4^ Biostatistics Collaboration Unit, Department of Biomedical Systems Informatics, Yonsei University College of Medicine, Seoul, South Korea

**Keywords:** breast reconstruction, deep inferior epigastric perforator flap, robot surgery, conventional DIEP, robotic DIEP, clinical outcome

## Abstract

**Background:**

A robotic deep inferior epigastric perforator (DIEP) flap created through a totally extraperitoneal approach minimizes violation of the donor site, which may lead to postoperative pain reduction and rapid recovery. The authors compared the clinical outcomes of robotic and conventional DIEP flap breast reconstructions.

**Methods:**

Data from consecutive patients who underwent mastectomy with DIEP flaps for breast reconstruction between July 2017 and January 2021 were retrospectively reviewed. Patients were divided into robotic and conventional DIEP groups, and the two groups were matched using the inverse probability of treatment weighting method. They were compared based on the reconstruction time, drainage amount, postoperative pain, rescue analgesics, hospital stay, complications, and BREAST-Q scores.

**Results:**

After matching, a dataset of 207 patients was formed, including 21 patients in the robotic DIEP group and 186 patients in the conventional DIEP group. The mean reconstruction time was longer in the robotic DIEP group than in the conventional DIEP group (*P*<0.001). In the robotic group, pain intensity during the postoperative 6–24 hours was significantly reduced (*P*=0.001) with less use of fentanyl (*P*=0.003) compared to the conventional DIEP group. The mean length of hospital stay for the robotic DIEP group was shorter than that for conventional DIEP (*P*=0.002). BREAST-Q scores indicated a higher level of the abdominal physical well-being domain in the robotic group (*P*=0.020). Complication rates were comparable between the two groups.

**Conclusions:**

This study suggests that a robotic DIEP flap offers enhanced postoperative recovery, accompanied by a reduction in postoperative pain and hospital stay.

## Introduction

As surgical techniques have improved and patient expectations have increased, the goal of breast reconstruction is to make breasts natural-looking and esthetically pleasing while minimizing patient morbidity. Autologous breast reconstruction using abdominal tissue has been developed to decrease donor-site morbidities. The deep inferior epigastric perforator (DIEP) flap has gained popularity since its introduction in 1989 and is currently the most commonly performed procedure to reduce the morbidity of the donor site (1–5). There is also the superficial inferior epigastric artery flap, which does not damage the rectus muscle and fascia at all, but its use is limited owing to the inconsistency of a reliable superficial inferior epigastric artery.

However, even during DIEP flap elevation, an incision in the anterior rectus fascia is inevitable. Especially when a reliable perforator is located near the umbilicus, an extensive incision over the fascia is needed. Dissection, splitting, and traction of the upper structures above the pedicle are required to reach the pedicle. These procedures may increase donor-site morbidities. These limitations can be overcome using minimally invasive approaches, such as robotic or laparoscopic approaches (6–10). They are used in the dissection of the pedicle coursing underneath the rectus muscle during harvesting of the DIEP flap. Therefore, violation of the anterior rectus fascia, rectus muscle, and motor nerves can be minimized compared with conventional DIEP flaps. Despite several reports of DIEP flap harvesting using robots, there is still a lack of data comparing the outcomes of robotic and conventional DIEP flaps for reconstruction.

Robotic DIEP flap harvest is expected to provide significant benefits in decreasing donor-site morbidity. This may lead to postoperative pain reduction, rapid recovery, and donor site well-being. This study aimed to perform a robotic DIEP flap harvesting through a totally extraperitoneal approach and compare the postoperative outcomes between robotic and conventional DIEP flap breast reconstruction.

## Materials and methods

### Study population

**Figure 1:**
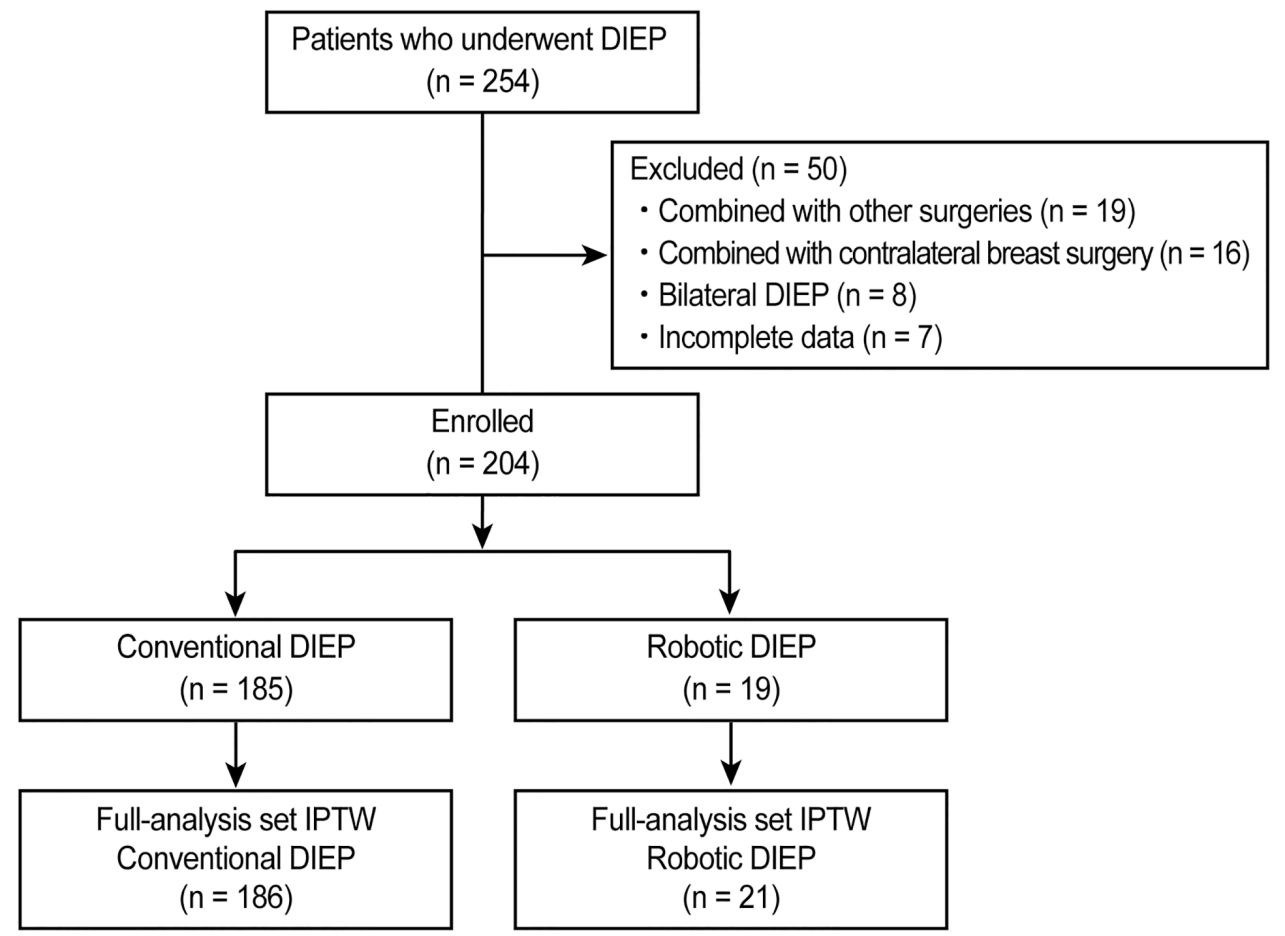
CONSORT flow diagram of patient selection. DIEP, deep inferior epigastric perforator; IPTW, inverse probability of treatment weighting.

This retrospective study was conducted at a single institution. Data from 254 consecutive Korean patients with breast cancer who underwent total mastectomy with immediate conventional DIEP flap or robotic DIEP flap breast reconstruction between July 2017 and January 2021 were identified from specified electronic medical records. To ensure uniformity in patient selection by reducing potential surgical confounding factors, 19 patients who underwent other simultaneous surgeries, 16 patients who underwent combined contralateral breast surgeries, 8 patients who underwent bilateral DIEP flap surgeries, and 7 patients with incomplete data were excluded. Finally, the remaining 204 patients who underwent unilateral DIEP flap breast reconstruction were eligible for the study and were classified into one of two groups: those who underwent conventional DIEP flap surgery (conventional DIEP, n = 185) and those who underwent robotic DIEP flap surgery (robotic DIEP, n = 19) (**Figure 1**). The robotic DIEP flap breast reconstruction was performed on patients who had single or closely grouped perforators with a short intramuscular course and consented to robotic surgery before surgery.

### Surgical techniques

**Figure 2:**
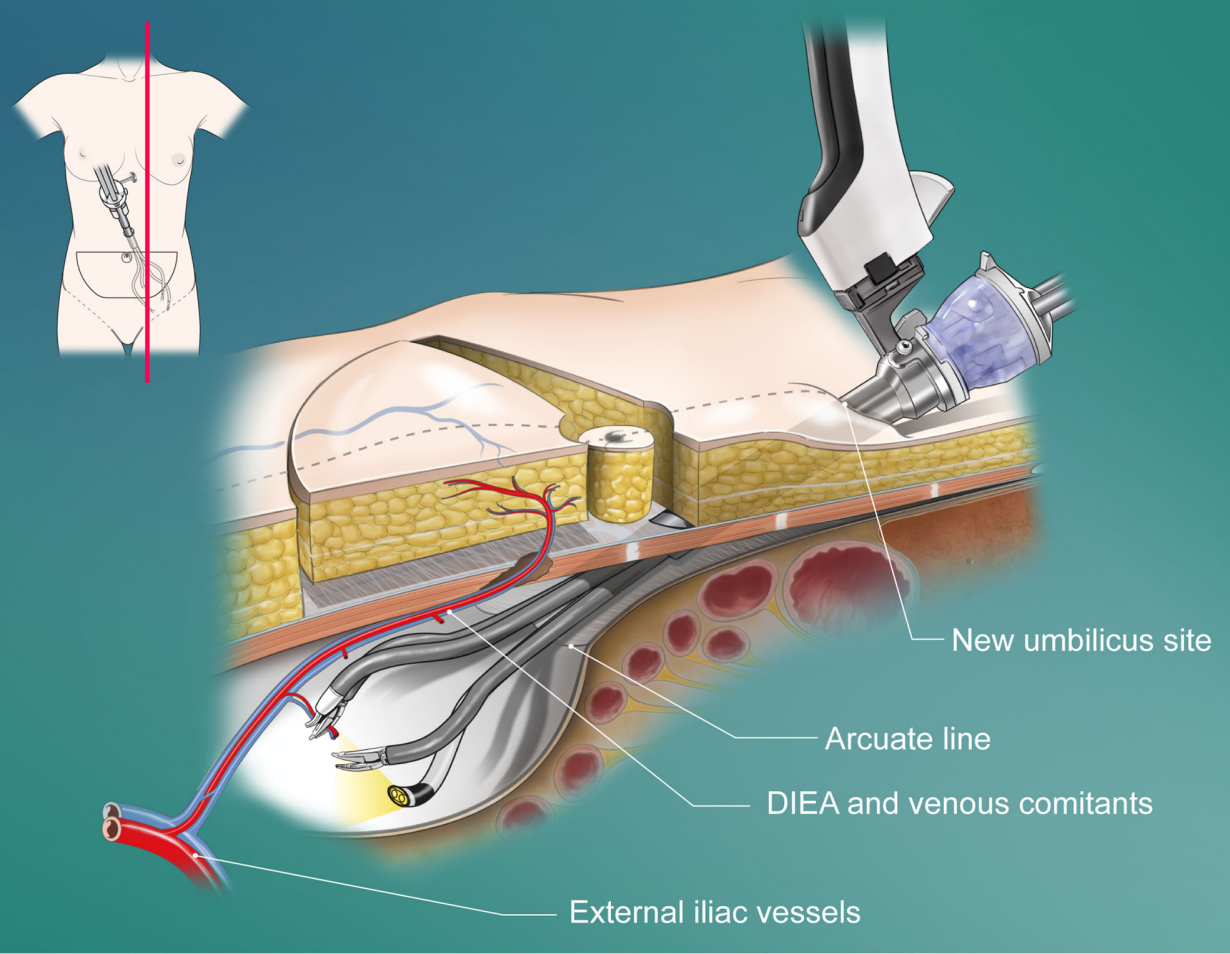
Schematic diagram of the penetrating placement of the single port in a robotic DIEP flap harvesting through a totally extraperitoneal approach. DIEA, deep inferior epigastric artery.

The conventional DIEP technique was performed in a standard manner by splitting the anterior rectus fascia and rectus abdominis muscles. The robotic DIEP technique was performed as previously reported by the authors (8). Briefly, in the robotic technique, the preselected perforator was dissected with the conventional method until the intramuscular course ended, followed by preperitoneal blunt dissection with a surgeon’s finger or balloon device (OMS-PDB1000; Covidien, Dublin, Ireland) through a 1.5-cm fascial incision on the linea semilunaris to secure the working space. The port was then inserted directly through the fascia into the preperitoneal space. When using a single-port robotic system (da Vinci SP; Intuitive Surgical, Sunnyvale, CA), the single port penetrates the new umbilicus site and fascia (**Figure 2**). The operation table was placed in the Trendelenburg position to avoid collision with the patient’s head or chest. Gas insufflation was maintained at 8 mmHg, and the robot was docked. Using the robot, the pedicle was dissected with ligation of all collateral vascular branches and divided near the origin (see Video, Supplemental Material 1, which shows the robotic dissection of the pedicle). After undocking the robot, the remaining attachments from the intramuscular portion were divided. Finally, the pedicle was delivered through a small fascial incision.

### Postoperative pain management

Before the end of the surgery, all patients received 1 μg/kg fentanyl (Hana Pharm, Seoul, Korea) and 0.3 mg of ramosetron (Nasea; Astellas Pharma Korea, Seoul, Korea) to control postoperative pain and postoperative nausea and vomiting (PONV). All patients received an intravenous (IV) patient-controlled analgesia (PCA) device (Anapa plus; E-HWA Biomedics, Seoul, Korea), programmed to 2 mL/h for background infusion, a demand volume of 0.5 mL, and a lock-out interval of 15 min, with a total volume of 100 mL. The PCA regimen comprised fentanyl and 0.3 mg of ramosetron, which were mixed with normal saline to achieve a total volume of 100 mL (11).

Data regarding postoperative pain were obtained from an electronic medical database that was recorded by a PCA management team comprising two qualified nurses. All eligible patients were informed on how to rate their pain intensity using the numerical rating scale (NRS; 0, no pain; 10, worst pain possible) in the pre-anesthetic room (12), after which they were moved to the post-anesthetic care unit (PACU) and had emerged from anesthesia. The recovery nurses assessed their NRS scores. The patients were instructed about the use of the PCA device and encouraged to push the button whenever they experienced pain. In patients who experienced sustained pain with a resting NRS score of ≥4, 50 µg IV fentanyl was administered as an additional rescue analgesic. After the patients were transferred to the admission room, postoperative NRS assessments were performed at 0−6, 6−24, and 24−48 h (11). In patients who suffered from prolonged pain with an NRS score of ≥4 in the admission room, 1 g IV paracetamol (Dai Han Pharm, Seoul, Korea), 30 mg ketorolac (Hana Pharm), 50 mg tridol (Yuhan. Co., Seoul, Korea), or 25 µg pethidine HCl (Jeil Pharm. Co. Ltd., Daegu, Korea) as an additional analgesic.

### Data collection and outcomes

Demographic, clinical, and laboratory data were collected from the electronic medical records. Demographic data included age, body mass index (BMI), American Society of Anesthesiologists (ASA) physical status classification, comorbidities (hypertension and diabetes mellitus), smoking history, menopausal status, and neoadjuvant chemotherapy. Oncologic characteristics, such as tumor pathology and pathological stage, were also evaluated. Data on intraoperative characteristics, operation times, blood loss, red blood cell transfusion, type of mastectomy, lymph node procedure, and specimen weight were collected. Postoperative variables, including drainage amount, laboratory values, length of hospital stay, postoperative adjuvant therapy, postoperative complications, postoperative pain, rescue analgesics, and PONV, were evaluated. Postoperative complications included hematoma, flap loss, infection, donor site wound problem, seroma, fat necrosis, and abdominal hernia (11). Furthermore, patient-reported outcomes were assessed using the BREAST-Q questionnaire. Patients included in this cohort were asked to complete the BREAST-Q questionnaire using a paper survey when visiting an outpatient clinic at least 6 months after the completion of reconstruction. The authors assessed the following domains: satisfaction with breasts, psychosocial well-being, physical well-being of the chest and abdomen, and satisfaction with the abdomen. Scores on the BREAST-Q domains ranged from 0 to 100, with higher scores indicating higher levels of satisfaction or improved well-being.

### Statistical analysis

Continuous variables were expressed as mean ± standard deviation and compared using Student’s *t*-test. Categorical variables are expressed as numbers (percentages) and compared using the chi-squared or Fisher’s exact test, depending on the size of the cell frequencies. Since data were retrospectively collected, the inverse probability of treatment weighting (IPTW) method was applied to adjust for confounding factors, including age, BMI, ASA physical status classification, hypertension, diabetes mellitus, smoking history, menopause, and neoadjuvant therapy (13). Logistic regression was used to regress the group variable on these confounding variables to calculate propensity scores (PSs). The goodness-of-fit of this logistic regression model was evaluated using the Hosmer–Lemeshow test (*P*=0.896). Moreover, 1/PS and 1/(1-PS) were weighed in the treatment and control groups, respectively. We stabilized and trimmed the weights to minimize the influence of extreme weights (14). To analyze the inverse probability of treatment-weighted data, we performed a t-test with the R command svyttest for continuous variables and the Rao-Scott Chi-square test for categorical variables with R command svychisq in the R package survey (the R Foundation). Statistical analysis was conducted using R version 4.0.4 (R Environment). Statistical significance was set at *P *< 0.05.

### Ethics

This study was performed in a single-center university hospital following approval from the Institutional Review Board (IRB) and the Hospital Research Ethics Committee of Severance Hospital, Yonsei University Health System, Seoul, Korea (IRB number: 4-2020-1397) and following the ethical standards of the current version of the Declaration of Helsinki. The need for prior consent was waived because of the retrospective nature of the anonymous data.

## Results

The patients` demographic characteristics are demonstrated in **Table 1**. After applying the IPTW method, a dataset with 207 patients was formed, including 21 patients in the robotic DIEP group and 186 patients in the conventional DIEP group. No significant differences were noted in demographic characteristics before and after IPTW adjustment.

**Table 1:** Demographic characteristics by using Inverse Probability of Treatment Weighting.

Variables	Before IPTW			After IPTW *		
	Conventional DIEP(N = 185)	Robotic DIEP(N = 19)	*P*–value	Conventional DIEP(N = 186)	Robotic DIEP(N = 21)	*P*–value
Age, year	48.6 ± 7.9	47.8 ± 5.7	0.663	48.5 ± 7.8	48.5 ± 6.6	0.998
BMI, kg/m^2^	24.0 ± 3.1	23.6 ± 3.5	0.631	23.9 ± 3.0	23.9 ± 3.6	0.942
ASA physical status			>.999			0.710
I	113 (61)	12 (63)		114 (61)	13 (63)	
II	65 (35)	7 (37)		66 (35)	8 (37)	
III	7 (4)	0 (0)		6 (3)	0 (0)	
Co-morbidities						
Hypertension	22 (12)	1 (5)	0.702	21 (11)	2 (7)	0.639
Diabetes	10 (5)	0 (0)	0.603	9 (5)	0 (0)	0.295
Smoking history			>.999			0.672
Non-smoker	178 (96)	19 (100)		179 (97)	21 (100)	
Ex-smoker	4 (2)	0 (0)		4 (2)	0 (0)	
Current smoker	3 (2)	0 (0)		3 (2)	0 (0)	
Postmenopausal status	46 (25)	2 (11)	0.255	44 (24)	4 (20)	0.768
Neoadjuvant chemotherapy	30 (16)	4 (21)	0.530	31 (17)	3 (15)	0.807
Tumor pathology			0.939			0.962
DCIS	53 (29)	6 (32)		52 (28)	5 (25)	
IDC	110 (60)	11 (58)		111 (60)	13 (62)	
Infiltrative other	22 (12)	2 (11)		22 (12)	3 (12)	
Stage			0.715			0.560
0	51 (28)	7 (37)		51 (27)	6 (28)	
1	71 (38)	8 (42)		71 (39)	11 (51)	
2	56 (30)	4 (21)		57 (30)	4 (20)	
3	7 (4)	0 (0)		7 (4)	0 (0)	

Values are mean ± standard deviation or number (%) of patients.

DIEP, deep inferior epigastric artery perforator; ASA, American Society of Anesthesiologists; BMI, body mass index; CEA, carcinoembryonic antigen; CA 15-3, cancer antigen 15-3; DCIS, ductal carcinoma in situ; IDC, invasive ductal carcinoma; IPTW, Inverse Probability of Treatment Weighting.

*Counts in the weighted data may not sum to expected totals owing to rounding. Percentage may not total 100 because of rounding, and disagreements between numbers and percentages in the weighted data are the result of rounding of non-integer number value.

A comparison of crude and IPTW-adjusted operative variables is presented in **Table 2**. The mean reconstruction time was significantly longer in the robotic DIEP group than in the conventional DIEP group (both P<0.001). The number of patients who underwent nipple-sparing mastectomy was significantly higher in the robotic DIEP group than in the conventional DIEP group. The specimen weights were not significantly different between the two groups. In addition, there were no differences in blood loss, patients transfused during surgery, lymph node procedure, or specimen weight between the two groups.

**Table 2:** Operative variables .

Variables	Before IPTW			After IPTW		
	Conventional DIEP(N = 185)	Robotic DIEP(N = 19)	*P*–value	Conventional DIEP(N = 186)	Robotic DIEP(N = 21)	*P*–value
Reconstruction time, min	438 ± 83	507 ±72	<.001*	438 ± 84	509 ± 71	<.001*
Blood loss, mL/hr	0.2 ± 0.2	0.3 ± 0.2	0.290	0.2 ± 0.2	0.3 ± 0.2	0.779
Intraoperative transfusion, n	13 (7)	2 (11)	0.636	13 (7)	2 (7)	0.986
Type of mastectomy			0.002*			0.006*
Nipple sparing	91 (49)	17 (90)		92 (49)	18 (87)	
Skin sparing	94 (51)	2 (10)		94 (51)	3 (13)	
Lymph node procedure						
SLNB then ALND	56 (30)	4 (21)	0.565	57 (31)	4 (20)	0.364
Specimen weight, g	548 ± 214	494 ± 181	0.291	546 ± 214	507 ± 177	0.361

Values are mean ± standard deviation or number (%) of patients. *P < 0.05.

DIEP, deep inferior epigastric artery perforator; RBC, red blood cell; SLNB, sentinel lymph node biopsy; ALND, axillary lymph node dissection; IPTW, Inverse Probability of Treatment Weighting.

In the 19 robotic surgeries, the mean intramuscular course of the pedicle was 4.1 cm, and the mean fascial incision length around the pedicle was 4.3 cm. The mean robot console time was 68.8 min. There was one case of open conversion in which the pedicle was ligated because the main pedicle was misrecognized as a side branch during the robotic surgery, and the pedicle on the opposite side was used. No peritoneal perforation or uncontrolled bleeding was observed.

The postoperative clinical and laboratory variables of the two groups are presented in **Table 3**. There was a significant difference in the amount of drainage from the donor site on postoperative day 0 (conventional group, 68 ± 29 mL vs. robot group, 55 ± 26 mL; P=0.031 after IPTW). Patients in the robotic DIEP group showed significantly lower white blood cell (WBC) count and neutrophil count on postoperative day 0 than those in the conventional DIEP group; however, no group difference in WBCs was observed after IPTW adjustment. Patients in the robotic DIEP group showed a significantly shorter postoperative hospital stay than those in the conventional DIEP group (7.92 ± 1.20 days vs. 8.77 ± 1.74 days, respectively; P=0.002 after IPTW). Other variables, including the complication rate, were comparable between the two groups.

**Table 3:** Postoperative variables and laboratory values.

Variables	Before IPTW			After IPTW			
	Conventional DIEP(N = 185)	Robotic DIEP(N = 19)	*P*–value		Conventional DIEP(N = 186)	Robotic DIEP(N = 21)	*P*–value
Drainage amounts from the donor site, mL							
POD 0	68 ± 29	58 ± 29	0.147		68 ± 29	55 ± 26	0.031*
POD 1	93 ± 29	95 ± 34	0.801		93 ± 29	93 ± 36	0.961
POD 2	81 ± 39	87 ± 31	0.472		81 ± 38	82 ± 30	0.832
Patients who received RBC transfusion, n							
POD 0	9 (5)	3 (16)	0.088		9 (5)	2 (11)	0.215
POD 1	7 (4)	1 (5)	0.549		7 (4)	1 (4)	0.940
POD 2	2 (1)	0 (0)	>.999		2 (1)	0 (0)	0.635
White blood cell count, 10^3^/µL							
Preoperative	5.8 ± 1.8	6.0 ± 1.9	0.657		5.8 ± 1.8	6.1 ± 1.8	0.519
POD 0	12.0 ± 2.8	10.4 ± 2.9	0.033*		12.0 ± 2.8	10.7 ± 2.6	0.061
POD 1	10.2 ± 3.0	9.3 ± 2.0	0.274		10.2 ± 3.0	9.5 ± 1.7	0.145
Neutrophil count, 10^3^/µL							
Preoperative	3.5 ± 1.5	3.7 ± 1.8	0.610		3.5 ± 1.5	3.7 ± 1.6	0.582
POD 0	10.0 ± 2.6	8.4 ± 2.6	0.022*		10.0 ± 2.6	8.5 ± 2.5	0.027*
POD 1	8.4 ± 2.6	7.6 ± 1.8	0.303		8.4 ± 2.6	7.8 ± 1.5	0.135
Lymphocyte count, 10^3^/µL							
Preoperative	1.8 ± 0.6	1.8 ± 0.6	0.891		1.8 ± 0.6	1.8 ± 0.6	0.706
POD 0	1.2 ± 0.5	1.2 ± 0.5	0.725		1.2 ± 0.5	1.3 ± 0.5	0.548
POD 1	1.0 ± 0.5	0.9 ± 0.3	0.437		1.0 ± 0.5	1.0 ± 0.4	0.676
Neutrophil-lymphocyte ratio							
Preoperative	2.2 ± 1.2	2.2 ± 1.6	0.912		2.2 ± 1.2	2.1 ± 1.5	0.922
POD 0	9.5 ± 4.8	8.6 ± 4.9	0.435		9.5 ± 4.7	7.9 ± 4.6	0.143
POD 1	9.5 ± 5.2	8.8 ± 3.4	0.619		9.5 ± 5.2	8.7 ± 3.3	0.388
Postop-hospital stay, day	8.78 ± 1.74	7.95 ± 1.22	0.044*		8.77 ± 1.74	7.92 ± 1.20	0.002*
Postoperative adjuvant treatment							
Radiation therapy, n	48 (26)	4 (21)	0.786		49 (27)	4 (18)	0.392
Chemotherapy, n	69 (37)	5 (26)	0.486		70 (38)	6 (27)	0.422
Hormonal therapy, n	121 (65)	13 (68)	0.992		122 (66)	15 (72)	0.605
Postoperative complications							
Flap loss	4 (2.2)	1 (5.3)	0.399		4 (2.3)	1 (3.8)	0.640
Fat necrosis	3 (1.6)	1 (5.3)	0.326		3 (1.7)	2 (9.4)	0.085
Donor-site hematoma	2 (1.1)	0 (0)	>.999		2 (1.1)	0 (0)	0.634
Donor-site seroma	2 (1.1)	0 (0)	>.999		2 (1.1)	0 (0)	0.634
Donor-site wound problem	12 (6.5)	0 (0)	0.608		12 (6.3)	0 (0)	0.244
Abdominal hernia	0 (0)	0 (0)	-		0 (0)	0 (0)	-

Values are mean ± standard deviation or number (%) of patients. *P < 0.05.

DIEP, deep inferior epigastric artery perforator; Postop, postoperative; POD, postoperative day; RBC, red blood cell; IPTW, Inverse Probability of Treatment Weighting.

**Figure 3:**
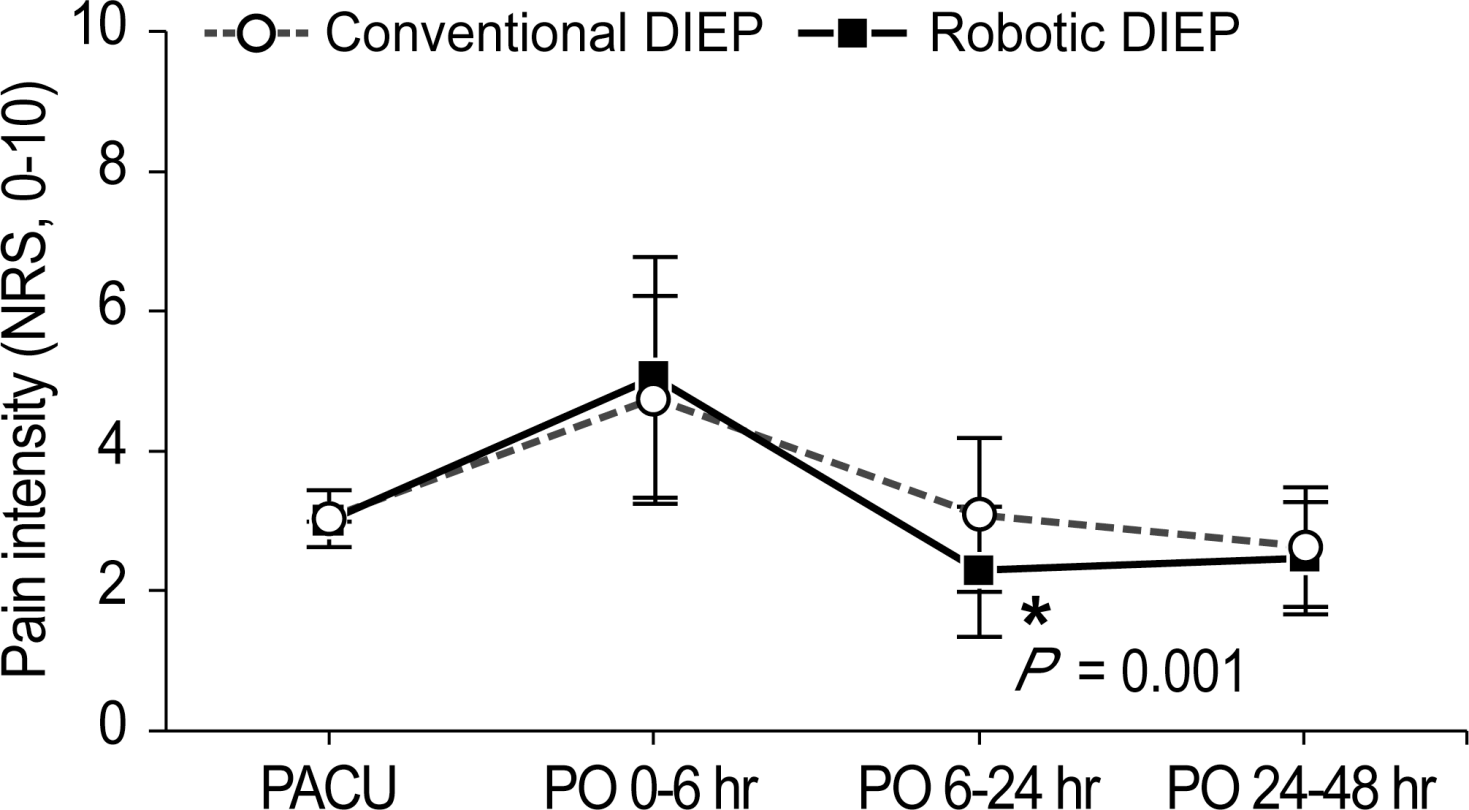
Pain intensity during the 48-h postoperative period. DIEP, deep inferior epigastric perforator; PACU, post-anesthesia care unit; PO, postoperative; NRS, numeric rating scale.

**Figure 3** illustrates the postoperative pain intensity in the two groups. The pain intensity at 0–6 h was the highest during the 48-h postoperative period. The pain intensity 6–24 h after surgery in the robotic DIEP group was significantly lower than that in the conventional DIEP group (2.3 ± 0.9 vs 3.1 ± 1.1, respectively; P=0.001), although a significantly lower dose of fentanyl in the PCA device was used in the robotic DIEP group. Furthermore, there were no differences between groups in the number of patients receiving other rescue analgesics, including paracetamol, ketorolac, and pethidine HCl, except for the number of patients receiving tridol during the 6–24 h postoperative period; no patients in the robotic DIEP group received tridol during the 6–24 h postoperative period, while about 20% of patients in the conventional DIEP group did (**Table 4**). There were no differences in the incidence or number of patients receiving antiemetics between groups.

**Table 4:** Postoperative nausea and vomiting, and analgesics profile.

Variables	Before IPTW				After IPTW		
	Conventional DIEP(N = 185)	Robotic DIEP(N = 19)	*P*–value	Conventional DIEP(N = 186)		Robotic DIEP(N = 21)	*P*–value
Fentanyl amounts in PCA, µg	1051 ± 483	853 ± 184	0.001*	1051 ± 490		851 ± 195	0.003*
Additional fentanyl in PACU, µg	13 ± 23	20 ± 28	0.211	13 ± 23		16 ± 26	0.614
Patients receiving paracetamol							
PO 0-6 hr	7 (4)	1 (5)	0.549	7 (4)		1 (5)	0.829
PO 6-24 hr	46 (25)	8 (42)	0.177	46 (25)		8 (38)	0.246
PO 24-48 hr	52 (28)	6 (32)	0.958	52 (28)		6 (30)	0.880
Patients receiving ketorolac							
PO 0-6 hr	179 (97)	19 (100)	>.999	180 (97)		21 (100)	0.411
PO 6-24 hr	181 (98)	19 (100)	>.999	182 (98)		21 (100)	0.500
PO 24-48 hr	166 (90)	19 (100)	0.227	167 (90)		21 (100)	0.135
Patients receiving tridol							
PO 0-6 hr	1 (1)	1 (5)	0.178	1 (1)		1 (3)	0.170
PO 6-24 hr	40 (22)	0 (0)	0.028*	40 (22)		0 (0)	0.023*
PO 24-48 hr	30 (16)	4 (21)	0.530	30 (16)		5 (24)	0.447
Patients receiving pethidine HCL							
PO 0-6 hr	1 (1)	0 (0)	>.999	1 (1)		0 (0)	0.737
PO 6-24 hr	13 (7)	0 (0)	0.616	13 (7)		0 (0)	0.221
PO 24-48 hr	8 (4)	0 (0)	>.999	8 (4)		0 (0)	0.341
Patients who PONV were experienced							
PACU	31 (17)	2 (11)	0.745	31 (17)		2 (9)	0.338
PO 0-6 hr	107 (58)	12 (63)	0.839	108 (58)		13 (61)	0.824
PO 6-24 hr	87 (47)	9 (47)	>.999	87 (47)		10 (46)	0.920
PO 24-48 hr	49 (27)	6 (32)	0.838	49 (27)		7 (32)	0.641
Patients receiving antiemetics							
PACU	31 (17)	2 (11)	0.745	31 (17)		2 (9)	0.335
PO 0-6 hr	17 (9)	2 (11)	0.692	17 (9)		3 (14)	0.516
PO 6-24 hr	6 (3)	0 (0)	>.999	6 (3)		0 (0)	0.409
PO 24-48 hr	5 (3)	1 (5)	0.448	5 (3)		1 (4)	0.786

Values are mean ± SD or number (%) of patients. *P < 0.05.

DIEP, deep inferior epigastric artery perforator; PONV, postoperative nausea and vomiting; PACU, post-anesthesia care unit; PO, postoperative; PCA, patient controlled analgesia; IPTW, Inverse Probability of Treatment Weighting.

In this cohort, 75 women (16 in the robotic DIEP group, 59 in the conventional DIEP group) completed the BREAST-Q. Patients in the robotic DIEP group had significantly higher scores for postoperative psychosocial well-being (77.7 ± 19.5 vs. 64.4 ± 16.1; P=0.007), physical well-being of the chest (73.9 ± 12.8 vs. 65.8 ± 12.9; P=0.028), and physical well-being of the abdomen (79.8 ± 13.6 vs. 71.4 ± 12.2; P=0.020) compared to those in the conventional DIEP group. There were no significant differences in the scores for satisfaction with the breasts and abdomen (Supplementary Material 2).

## Discussion

Among the various breast reconstruction techniques, the DIEP flap is known as one of the most advanced procedures because the abdominal rectus muscle areas are not harvested and thus has the advantage of minimizing morbidities in the donor site areas, which leads to an increased level of satisfaction (1–3, 15, 16)., However, during DIEP flap elevation, anterior rectus fascia, rectus muscle, and motor nerve violations can potentially occur (7, 17). To overcome this problem, Hivelin et al. harvested a DIEP flap with a totally extraperitoneal approach using a laparoscope (9); Gunclapalli et al. (6) and Selber (7) reported the use of a transabdominal preperitoneal approach with a multiport robotic system. Subsequently, the study’s senior author (DWL) introduced a robotic DIEP flap harvest through a totally extraperitoneal approach with a single-port robotic system (8). Although a totally extraperitoneal approach is less invasive compared to the transabdominal preperitoneal approach that penetrates the peritoneum, it has a steep learning curve owing to the narrow preperitoneal space. He indicated that a single-port robot optimized for narrow surgical spaces permits DIEP flap harvesting using a totally extraperitoneal approach. In recent years, reports of minimally invasive procedures for the methodological part of robotic DIEP flaps for breast reconstruction have increased, while reports of postoperative prognosis are still lacking.

There has been an increased focus in studies examining the enhanced recovery after surgery for patients with breast cancer after breast reconstruction (18–20). Since the development and implementation of early postoperative recovery in gastrointestinal surgery has been shown to improve perioperative outcomes and decrease the length of hospitalization, such protocols have been extended to patients with a wide variety of surgical diseases in an effort to enhance early postoperative recovery (21). Postoperative length of stay is commonly employed as an outcome measure for early postoperative recovery and serves as an indicator of functional recovery and return to normal activity, which is the ultimate aim of early postoperative recovery (18). Meanwhile, the absolute number of hospital days in the current study seems to be longer than that for microvascular breast reconstruction in the United States. According to Holoyda et al. (22), the mean length of hospital stay in the U.S. was 3.90 days in 2018. However, a direct comparison of hospital stays between two countries with different healthcare systems is not appropriate. In this study, postoperative hospital stay was significantly shorter in the robotic DIEP group than in the conventional DIEP group, which was consistent with the reports regarding robotic procedures in other types of surgery (23, 24). Flap-based reconstruction is one of the surgeries with the highest morbidities and longest hospital stays within the field of plastic and reconstructive surgery; in these fields, it is clinically significant that robotic DIEP can shorten the postoperative hospital stay by one day.

This study compared the effects of robotic DIEP flap breast reconstruction with those of conventional DIEP on postoperative pain intensity. There were no significant differences in postoperative NRS scores at 0–6 h; however, patients in the robotic DIEP group showed significantly lower NRS scores during the 6–24 h postoperative period (2.3 ± 0.9 vs. 3.1 ± 1.1, respectively; *P*=0.001). The amount of fentanyl mixed in the PCA device was significantly lower in the robotic DIEP group (851 ± 195 µg) than in the conventional DIEP group (1051 ± 490 µg) (P=0.003). However, the morphine equivalent dose of fentanyl mixed with PCA in the conventional DIEP group was higher than that in previous studies (25, 26), which may be the reason why there was not much of a difference in NRS scores, despite the statistically significant differences in the dose of fentanyl. Furthermore, the fentanyl amounts in the PACU and other rescue analgesics in the admission room were comparable between the two groups, while a significant difference in the number of patients receiving tridol during the 6-24 h postoperative period was noted. Postoperative pain has been reported to interfere with early postoperative recovery and cause chronic pain after surgery, which reduces physical activity and quality of life (27, 28). Such significant attenuation in NRS during the 6–24 h postoperative period may have contributed to shortening the postoperative hospital stay in robotic DIEP (29, 30).

Significant group differences were observed in operation time. The results showed that the duration of the robotic DIEP operation was significantly longer than that of the conventional DIEP procedure, which is consistent with the findings of other types of robotic surgery (11, 31, 32). This longer operative time is thought to be due to the additional time for preparation of the robot and a relatively longer robotic dissection time compared to conventional surgery (33). In the current study, the mean robot console time was 68.8 min and showed a decreasing pattern over time (data not shown). In addition to the longer operation time, another common drawback in the use of robotics is the cost (34). However, to claim that robotic surgery is expensive, a cost-effectiveness analysis is required. As robotic surgery becomes more popular, there have been discussions about the high cost of robotic surgery and its effectiveness. Although there is a report that states that robot-assisted radical prostatectomy has a higher cost compared to the open and laparoscopic approach, with relatively fewer health benefits (35), many studies predict that the high cost would be balanced by favorable clinical outcomes, such as a reduction in blood transfusion requirement, hospital stay, and perioperative complications (36–39). In addition, robotic thymectomy was associated with a lower total hospital cost than that with open surgery since it reduced the duration of intensive care unit and hospital stay (40). The increase in the number of robotic surgeries may lead to a significant reduction in the future operation time and better postoperative outcomes (41). The presence of more experienced professionals and optimal teamwork have caused a reduction in the operation time and led to cost-effectiveness, with more experienced centers having lower costs (41–43).

This study had a few limitations. First, the data were retrospectively collected from a single center, primarily from Korean patients. It is difficult to generalize these results to patients from different ethnic backgrounds or those treated under different institutional conditions. Second, the sample size was small, especially in patients who underwent robotic DIEP surgery, which may have contributed to the higher incidence of postoperative complications in the conventional DIEP group without a statistically significant difference. Therefore, to add clinical significance to the existing literature, further large-scale prospective controlled trials are required, especially with a greater number of samples of robotic DIEP surgery. However, this study provides evidence for future prospective trials in terms of reconstruction outcomes, such as donor site morbidity, enhanced recovery after surgery, and functional restoration at the donor site.

In conclusion, this is the first study to compare the effects of robotic DIEP flap breast reconstruction with those of conventional DIEP reconstruction on the postoperative clinical outcomes. We demonstrated that robotic DIEP flap breast reconstruction offers enhanced postoperative recovery, which was accompanied by attenuated pain intensity and reduced postoperative hospital stay. Furthermore, a significantly superior abdominal physical well-being score on patient-reported outcomes was noted in patients who underwent robotic DIEP flap breast reconstruction.

## Data availability statement

The original contributions presented in the study are included in the article/**Supplementary Material**. Further inquiries can be directed to the corresponding authors.

## Ethics statement

The studies involving human participants were reviewed and approved by Institutional Review Board and the Hospital Research Ethics Committee of Severance Hospital, Yonsei University Health System, Seoul, Korea. Written informed consent for participation was not required for this study in accordance with the national legislation and the institutional requirements.

## Author contributions

ML and JW, designed this project, data collection, processing, and manuscript writing. SS, HP, and JK, data collection, and review. HS and YK, data analysis, and processing. DL and NK, designed this project, interpretation, manuscript writing, review and editing. All authors contributed to the article and approved the submitted version.

## Conflict of interest

The authors declare that the research was conducted in the absence of any commercial or financial relationships that could be construed as a potential conflict of interest.

## Publisher’s note

All claims expressed in this article are solely those of the authors and do not necessarily represent those of their affiliated organizations, or those of the publisher, the editors and the reviewers. Any product that may be evaluated in this article, or claim that may be made by its manufacturer, is not guaranteed or endorsed by the publisher.
