# Supplementary material for: Clinical outcomes following robotic versus conventional DIEP flap in breast reconstruction: A retrospective matched study
Source: Front Oncol. 2022 Sep 14;12:989231. doi: 10.3389/fonc.2022.989231 (PMC9515388; doi:10.3389/fonc.2022.989231)
Supplement: Supplementary file 1 [file Table_1.docx]

Supplementary table 1. Postoperative BREAST-Q scores

| variables | Conventional DIEP  (N = 59) | Robotic DIEP  (N = 16) | *P*–value |
| --- | --- | --- | --- |
| Satisfaction with breasts | 56.2 ± 13.3 | 62.9 ± 17.1 | 0.101 |
| Psychosocial score | 64.4 ± 16.1 | 77.7 ± 19.5 | 0.007* |
| Physical well-being score, chest | 65.8 ± 12.9 | 73.9 ± 12.8 | 0.028* |
| Physical well-being score, abdomen | 71.4 ± 12.2 | 79.8 ± 13.6 | 0.020* |
| Satisfaction with abdomen^a^ | 7.3 ± 1.8 | 7.6 ± 2.1 | 0.520 |

Values are mean ± SD. * P <0.05

a, scores on the satisfaction with abdomen domain ranged from 0 to 12.
